# Adherence to Life’s Essential 8 is associated with lower risk of hyperuricemia among adults in Beijing, China

**DOI:** 10.3389/fnut.2025.1738146

**Published:** 2026-01-12

**Authors:** Rui Deng, Jiali Duan, Bo Yu, Yan Zhang, Yihong Yao, Rui Ma, Yimei Sha

**Affiliations:** Institute of Nutrition and Food Hygiene, Beijing Center for Disease Prevention and Control, Beijing, China

**Keywords:** adults, cardiovascular health, Chinese Nutrition and Health Surveillance, hyperuricemia, Life’s Essential 8

## Abstract

**Background:**

Hyperuricemia is linked to various cardiovascular diseases, yet evidence on the association of Life’s Essential 8, a newly updated metric for cardiovascular health, with hyperuricemia in the general Chinese population remains scarce.

**Methods:**

Data were obtained from 1,519 adults in Beijing as part of the Chinese Nutrition and Health Surveillance 2022. Logistic regression and restricted cubic spline models were used to assess the associations of Life’s Essential 8 score and its subscales with the risk of hyperuricemia. Stratified analyses by key demographic variables were also performed.

**Results:**

The prevalence of hyperuricemia was 13.76%. For every 10-unit increase in Life’s Essential 8 score and its subscale of health factor scores, the risk of hyperuricemia decreased by 24% (OR = 0.76, 95% CI = 0.66 ~ 0.87) and 23% (OR = 0.77, 95% CI = 0.70 ~ 0.84), respectively. Participants with high Life’s Essential 8 scores had a lower risk of hyperuricemia compared with those with low Life’s Essential 8 scores (OR = 0.32, 95% CI = 0.14 ~ 0.74), and similar results were found for those with high health factor scores (OR = 0.29, 95% CI = 0.17 ~ 0.49). These associations were more pronounced among participants who had high educational levels and middle-class annual household incomes.

**Conclusion:**

Our study revealed a negative association of the Life’s Essential 8 score with the risk of hyperuricemia in the general population of Beijing, China, which was also observed for its health factor subscale, highlighting the promise of adherence to the Life’s Essential 8 to maintain cardiovascular health and thereby reduce the disease burden of hyperuricemia.

## 1 Introduction

Hyperuricemia is a condition characterized primarily by elevated blood uric acid levels and is typically diagnosed when uric acid levels exceed 416.5 μmol/L in men and 357.0 μmol/L in women (1). Data from the National Health and Nutrition Examination Survey in the United States revealed that the prevalence of hyperuricemia remained relatively stable at around 20% from 2007 to 2016 (2). However, in Chinese adults, a study reported that the overall prevalence of hyperuricemia significantly increased from 11.1% (10.3%~11.8%) in 2015~2016 to 14.0% (13.1%~14.8%) in 2018~2019 (3). Given that high levels of blood uric acid are the main cause of gout, 36% of patients with hyperuricemia ultimately developed gout (4). In addition to gout, hyperuricemia is also associated with several diseases, such as metabolic syndrome (5), hypertension (6), diabetes (7) and kidney diseases (8).

Evidence has shown that adherence to healthy lifestyles can reduce serum uric acid levels by 70 ~ 90 μmol/L and the risk of hyperuricemia incidence by 41% (9, 10). However, the definitions of lifestyle components varied widely across these studies. Furthermore, hyperuricemia has been established as a risk factor for cardiovascular diseases (8). For instance, evidence from meta-analyses showed that hyperuricemia was associated with 22 and 33% greater risks of stroke incidence and mortality, respectively (11), and 41% increases in the risk of incident hypertension (12). These findings underscored the importance of maintaining cardiovascular health in its prevention. In 2020, to improve the cardiovascular health (CVH) of populations, the American Heart Association formulated an accessible and actionable definition of lifestyle for individuals, researchers and practitioners, called Life’s Simple 7 (13), which includes seven components: dietary quality, physical activity, exposure to cigarette smoking, body mass index status, and blood pressure, blood glucose and blood lipid levels. After 12 years of practice and application of Life’s Simple 7, the American Heart Association updated the definition and approach for CVH measurement in 2022 (14), named Life’s Essential 8 (14). This update added sleep health as a new metric and used a more comprehensive scoring algorithm to capture interindividual differences more sensitively than Life’s Simple 7.

Therefore, exploring whether higher Life’s Essential 8 scores (LES) contribute to a lower risk of hyperuricemia is necessary and valuable. Nevertheless, limited studies have detected this association, and existing research evidence predominantly derived from adults in United States (15–18). These studies collectively reported a significant negative relationship between LES and hyperuricemia, indicating that participants with high LES had 50 to 80% lower odds of hyperuricemia. For Chinese population, only two existing studies examined this association and showed similar results, but were conducted among elderly or multiethnic groups (19, 20), with little evidence regarding Chinese adults in the general population.

In this study, we aimed to identify the newly updated LES and explore its association with the risk of hyperuricemia in the general population in Beijing, China, with a focus on efforts for hyperuricemia prevention and related strategy formulation.

## 2 Materials and methods

### 2.1 Data source

**Figure 1:**
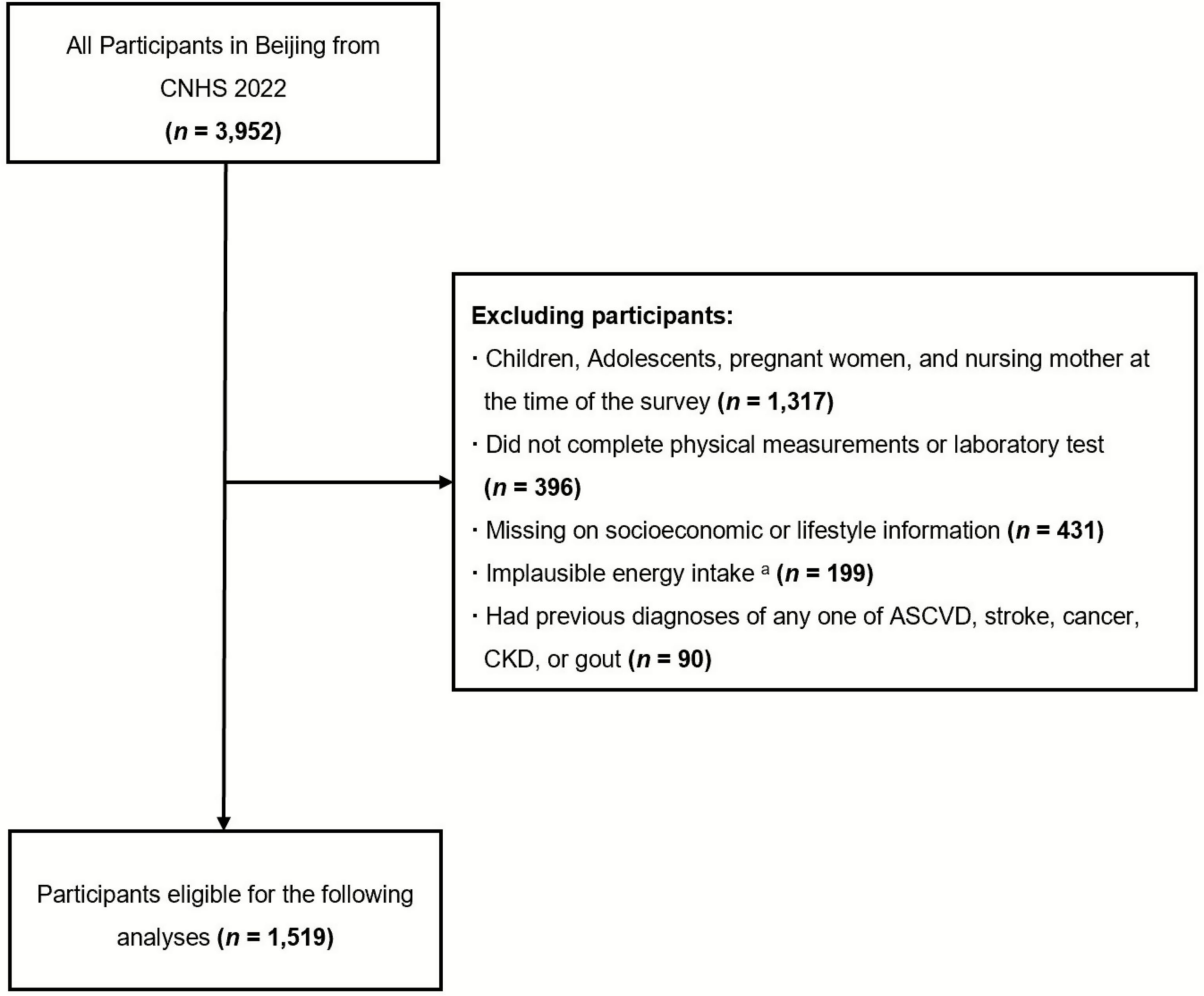
Flow chart of participant selection. Abbreviations: CNHS, China Nutrition and Health Surveillance; ASCVD, atherosclerosis cardiovascular disease; CKD, chronic kidney disease. ^a^Implausible daily energy intake was defined as <500 or >5,000 kcal/d.

Data were obtained from the Beijing subset of the Chinese Nutrition and Health Surveillance (CNHS) in 2022. The CNHS is a nationally representative cross-sectional study in mainland China. Using a multistage, stratified, and random sampling method, residents from 31 provinces were surveyed. Detailed information on the study protocol, sampling strategy, methodology, and quality assurance procedures can be found elsewhere (21). We utilized data from Beijing, which included a total of 3,952 participants, as the study population. Eligible participants were adults aged above 18 years who had plausible energy intake, complete data on physical measurements, laboratory tests, socioeconomic and lifestyle information, and an absence of any previously diagnosed serious diseases. Finally, 1,519 participants were included in this study. The process of sample selection is shown in Figure 1. The CNHS survey was approved by the Ethical Committee of the Chinese Center for Disease Control and Prevention (No: 201614). All the participants signed informed consent before the survey.

### 2.2 Indicators

#### 2.2.1 The Life’s Essential 8 score (LES)

The LES was calculated in accordance with the definitions and algorithms provided by the American Heart Association in 2022 (14). There are eight indicators in the LES, each with a score range of 0~100 points, comprising diet, physical activity, sleep, nicotine exposure, body mass index (BMI), blood glucose, blood pressure and non-HDL-C. Dietary intake was assessed via a food frequency questionnaire, and diet quality was evaluated via the Dietary Approaches to Stop Hypertension (DASH) diet score. The DASH diet score is the sum of eight component scores, each corresponding to a specific food domain: fruits, vegetables, nuts and legumes; low-fat dairy products; whole grains; sodium; sweetened beverages; and red and processed meats (22). For the first five components (fruits to whole grains), participants in the highest quintile received 5 points, and those in the lowest quintile received 1 point. For the remaining components (sodium to red and processed meats), this scoring was reversed (highest quintile = 1 point, lowest quintile = 5 points). The total score ranged from 8 to 40, with higher scores indicating greater adherence to the DASH diet. Finally, on the basis of the 90th, 75th, 50th, and 25th percentiles of the DASH score (22), participants were assigned scores of 100, 80, 50, 25, and 0 for the LES, respectively. Physical activity was defined as self-reported minutes of moderate or vigorous physical activity per week. According to the scoring criteria of LES, participants with more than 150 min of moderate or vigorous physical activity per week received 100 points, with a deduction of 10 points for each subsequent 30-min decrease (23). Sleep health was defined as self-reported average hours of sleep with 7~9 h earning the maximum score. Nicotine exposure included self-reported use of cigarettes and second-hand smoke exposure, with never smoking considered ideal. BMI was calculated as body weight (kilogram, kg) divided by height squared (meter, m). Blood glucose was defined as levels of plasma hemoglobinA1c, with a self-reported history of diabetes from the questionnaire used as a supplementary criterion. Blood pressure was measured via auscultatory method, with systolic and diastolic pressures defined by Korotkoff sounds. Three consecutive readings were taken for each participant, and the mean of these measurements was used for analyses. Non-HDL-C was defined as total cholesterol minus high-density lipoprotein cholesterol. Specific scoring details for calculating LES for each metric could be found elsewhere (14).

The overall LES was calculated as the unweighted average of all eight metric scores. LESs were classified as follows: high (80~100 points), indicating high CVH; moderate (50~79 points), indicating moderate CVH; and low (0~49 points), indicating low CVH. With the same calculation method and cutoff points, the subscales of health behaviors scores (HBS) was defined as the average of diet, physical activity, sleep and nicotine exposure, and health factor scores (HFS) was defined as that of BMI, blood glucose, blood pressure and non-HDL-C.

#### 2.2.2 Diagnosis of hyperuricemia

Fasting venous blood samples were collected during physical examinations, and serum uric acid levels were measured via the enzymatic kinetic method on a Randox/Hitachi 7600 automated analyzer. Hyperuricemia was diagnosed as a serum uric acid level >420 μmol/L for males and >360 μmol/L for females.

### 2.3 Covariates

The covariates in this study consisted of demographic information (age, sex, living area, ethnicity, education, income, and marital status) assessed through standardized questionnaires, and the estimated glomerular filtration rate (eGFR) derived from the CKD-EPI creatinine equation using serum creatinine, age, sex, and race. Age was categorized into three groups (18~44 years, 45~59 years and above 60 years). Educational level was classified as junior high school or below, high school, or college or above. Annual household income per capita was categorized into three groups using thresholds of 20,000 Chinese Yuan (CNY) and 50,000 CNY based on the definition in previous study (24), as well as sample balance and the economic conditions in the capital region.

### 2.4 Statistical analysis

Categorical variables were presented as numbers with percentages (%), and continuous variables were presented as the means and standard deviations. Analysis of variance was used to detect the characteristic differences between the LES groups. A logistic model was fitted to analyse the relationship of the LES groups and every 10-point increase in the LES with the risk of hyperuricemia. To explore whether a nonlinear relationship existed between the LES and the risk of hyperuricemia, the restricted cubic spline (RCS) model was adopted on the basis of five knots (5th, 25th, 50th, 75th, and 95th). We performed stratified analyses by key demographic variables, including age group, sex, living area, educational level, and household income. The significance of the interaction effects was assessed via the *p* value derived from the product terms between the LES and these stratification factors. The aforementioned analyses were repeated for the HBS and HFS subscales. All analyses were performed via SAS version 9.4, with statistical significance defined as a two-sided *p* value <0.05.

## 3 Results

### 3.1 Basic characteristics

Among the 1,519 participants, 679 (44.70%) were males, and most (62.87%) lived in urban areas (Table 1). The average level of uric acid was 302.78 μmol/L, and the prevalence of hyperuricemia was 13.76%. The high LES, HBS and HFS accounted for 45.04%, 44.85%, and 54.23%, respectively. Those who were younger, lived in urban areas, had a college degree or above and had an annual income of more than 50,000 CNY/year were more likely to have high LES and HFS.

**Table 1:** Basic characteristics of the participants.

Characteristic	Overall^d^	LES			HBS			HFS		
		Low	Moderate	High	Low	Moderate	High	Low	Moderate	High
Overall^d^		43 (2.83)	953 (62.74)	523 (34.43)	89 (5.86)	710 (46.74)	720 (47.40)	140 (9.22)	798 (52.53)	581 (38.25)
Age group^a^^,^^c^										
18~	544 (35.81)	13 (30.23)	286 (30.01)	245 (46.85)	29 (32.58)	271 (38.17)	244 (33.89)	29 (20.71)	220 (27.57)	295 (50.77)
45~	457 (30.09)	13 (30.23)	303 (31.79)	141 (26.96)	28 (31.46)	199 (28.03)	230 (31.94)	48 (34.29)	261 (32.71)	148 (25.47)
60~	518 (34.10)	17 (39.53)	364 (38.20)	137 (26.20)	32 (35.96)	240 (33.80)	246 (34.17)	63 (45.00)	317 (39.72)	138 (23.75)
Sex^a^^,^^b^										
Males	679 (44.70)	36 (83.72)	503 (52.78)	140 (26.77)	79 (88.76)	387 (54.51)	213 (29.58)	72 (51.43)	366 (45.86)	241 (41.48)
Females	840 (55.30)	7 (16.28)	450 (47.22)	383 (73.23)	10 (11.24)	323 (45.49)	507 (70.42)	68 (48.57)	432 (54.14)	340 (58.52)
Living area^a^^,^^b^^,^^c^										
Urban	955 (62.87)	20 (46.51)	554 (58.13)	381 (72.85)	44 (49.44)	419 (59.01)	492 (68.33)	61 (43.57)	472 (59.15)	422 (72.63)
Rural	564 (37.13)	23 (53.49)	399 (41.87)	142 (27.15)	45 (50.56)	291 (40.99)	228 (31.67)	79 (56.43)	326 (40.85)	159 (27.37)
Ethnicity										
Han	1,452 (95.59)	40 (93.02)	910 (95.49)	502 (95.98)	83 (93.26)	680 (95.77)	689 (95.69)	132 (94.29)	760 (95.24)	560 (96.39)
Non-Han	67 (4.41)	3 (6.98)	43 (4.51)	21 (4.02)	6 (6.74)	30 (4.23)	31 (4.31)	8 (5.71)	38 (4.76)	21 (3.61)
Educational level^a^^,^^c^										
Junior school or below	608 (40.03)	18 (41.86)	432 (45.33)	158 (30.21)	34 (38.20)	310 (43.66)	264 (36.67)	75 (53.57)	369 (46.24)	164 (28.23)
High school	362 (23.83)	15 (34.88)	237 (24.87)	110 (21.03)	25 (28.09)	160 (22.54)	177 (24.58)	35 (25.00)	194 (24.31)	133 (22.89)
College or above	549 (36.14)	10 (23.26)	284 (29.80)	255 (48.76)	30 (33.71)	240 (33.8)	279 (38.75)	30 (21.43)	235 (29.45)	284 (48.88)
Household income per capita (CNY/year)^a^^,^^c^										
<20,000	384 (25.58)	17 (39.53)	276 (28.96)	91 (17.40)	29 (32.58)	200 (28.17)	155 (21.53)	55 (39.29)	223 (27.94)	106 (18.24)
20,000~	565 (37.20)	14 (32.56)	348 (36.52)	203 (38.81)	28 (31.46)	260 (36.62)	277 (38.47)	47 (33.57)	301 (37.72)	217 (37.35)
50,000~	570 (37.52)	12 (27.91)	329 (34.52)	229 (43.79)	32 (35.96)	250 (35.21)	288 (40.00)	38 (27.14)	274 (34.34)	258 (44.41)
Marital status										
Married	1,325 (87.23)	38 (88.37)	841 (88.25)	446 (85.28)	79 (88.76)	622 (87.61)	624 (86.67)	129 (92.14)	707 (88.60)	489 (84.17)
Other status	194 (12.77)	5 (11.63)	112 (11.75)	77 (14.72)	10 (11.24)	88 (12.39)	96 (13.33)	11 (7.86)	91 (11.40)	92 (15.83)
Hyperuricemia^a^^,^^c^										
No	1,310 (86.24)	34 (79.07)	801 (84.05)	475 (90.82)	79 (88.76)	598 (84.23)	633 (87.92)	111 (79.29)	672 (84.21)	527 (90.71)
Yes	209 (13.76)	9 (20.93)	152 (15.95)	48 (9.18)	10 (11.24)	112 (15.77)	87 (12.08)	29 (20.71)	126 (15.79)	54 (9.29)
LES^a^^,^^b^^,^^c^	74.28 (11.91)	44.56 (3.73)	68.73 (7.41)	86.84 (4.94)	55.84 (9.15)	69.47 (9.53)	81.3 (9.25)	56.04 (8.49)	70.84 (8.47)	83.4 (8.7)
HBS^a^^,^^b^^,^^c^	75.43 (15.28)	47.33 (12.54)	70.67 (14.15)	86.41 (8.61)	40.62 (7.06)	66.96 (8.44)	88.09 (5.94)	72.13 (15.58)	74.83 (15.05)	77.05 (15.36)
HFS^a^^,^^b^^,^^c^	73.13 (16.86)	41.8 (12.34)	66.79 (14.22)	87.26 (9.53)	71.05 (17.17)	71.99 (16.66)	74.51 (16.92)	39.96 (7.04)	66.85 (8.22)	89.75 (6.51)
Uric acid (μmol/L)^a^^,^^c^	302.78 (84.21)	335.21 (98.23)	313.79 (85.55)	280.04 (75.31)	320.15 (83.55)	311.27 (88.37)	292.25 (78.73)	326.7 (92.11)	310.14 (83.74)	286.9 (80.16)
eGFR (ml/min/1.73^2^)^a^^,^^b^^,^^c^	99.91 (15.87)	97.72 (17.23)	99.11 (16.14)	101.54 (15.14)	99.01 (15.56)	100.37 (16.73)	99.56 (15.02)	97.01 (16.11)	98.23 (15.78)	102.91 (15.48)

CNY, Chinese Yuan; LES, Life’s Essential 8 score; HBS, healthy behaviors score; HFS, healthy factors score; eGFR, estimated glomerular filtration rate.

a The corresponding characteristic had significant differences between LES groups.

b The corresponding characteristic had significant differences between HBS groups.

c The corresponding characteristic had significant differences between HFS groups.

d Values of polytomous may not sum to 100% due to rounding.

### 3.2 LES, HBS, HFS and the risk of hyperuricemia

**Figure 2:**
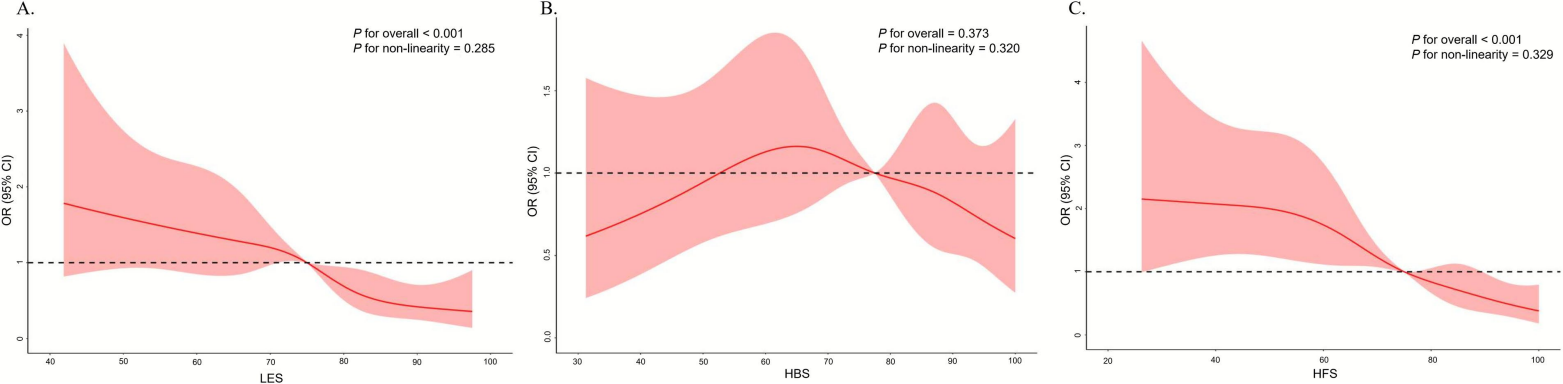
RCSs of the associations between LES **(A)**, HBS **(B)**, and HFS **(C)** and the risk of hyperuricemia. LES, life’s essential 8 score; HBS, health behavior score; HFS, health factor score. ^a^Based on 5-knot (5th, 25th, 50th, 75th, 95th), RCS models adjusted for age, sex, living area, ethnicity, educational level, household income, marital status, and eGFR.

As presented in Table 2, for every 10-unit increase in LES and HFS, the risk of hyperuricemia decreased by 24% (OR = 0.76, 95% CI = 0.66~0.87) and 23% (OR = 0.77, 95% CI = 0.70~0.84), respectively, in the fully adjusted model. Participants with a high LES had a lower risk of hyperuricemia than those with a low LES did (OR = 0.32, 95% CI = 0.14~0.74), and a similar result was found in those with high HFS (OR = 0.29, 95% CI = 0.17~0.49). However, no significant association was found between HBS and the risk of hyperuricemia. Additionally, RCS analysis did not reveal a significant nonlinear relationship between LES, HBS or HFS and the risk of hyperuricemia (Figure 2).

**Table 2:** The associations between LES, HBS, HFS and the risk of hyperuricemia.

CVH score	Model 1^a^		Model 2^b^		Model 3^c^	
	OR (95% CI)	*p* value^d^	OR (95% CI)	*p* value	OR (95% CI)	*p* value
LES						
Low	Reference	<0.001	Reference	<0.001	Reference	<0.001
Moderate	0.72 (0.34, 1.53)		0.72 (0.33, 1.54)		0.70 (0.32, 1.54)	
High	0.38 (0.17, 0.84)		0.36 (0.16, 0.83)		0.32 (0.14, 0.74)	
Per 10-unit increase	0.80 (0.71, 0.90)	<0.001	0.77 (0.68, 0.88)	<0.001	0.76 (0.66, 0.87)	<0.001
HBS						
Low	Reference	0.223	Reference	0.569	Reference	0.583
Moderate	1.48 (0.74, 2.94)		1.61 (0.80, 3.23)		1.64 (0.80, 3.34)	
High	1.09 (0.54, 2.18)		1.27 (0.62, 2.61)		1.28 (0.61, 2.69)	
Per 10-unit increase	0.93 (0.85, 1.02)	0.123	0.96 (0.87, 1.06)	0.447	0.96 (0.87, 1.07)	0.473
HFS						
Low	Reference	<0.001	Reference	<0.001	Reference	<0.001
Moderate	0.72 (0.46, 1.13)		0.67 (0.42, 1.06)		0.64 (0.40, 1.03)	
High	0.39 (0.24, 0.64)		0.32 (0.19, 0.54)		0.29 (0.17, 0.49)	
Per 10-unit increase	0.82 (0.76, 0.90)	<0.001	0.79 (0.72, 0.86)	<0.001	0.77 (0.70, 0.84)	<0.001

CVH, cardiovascular health; LES, Life’s Essential 8 score; HBS, healthy behaviors score; HFS, healthy factors score; eGFR, estimated glomerular filtration rate.

a Model 1 was crude model without any adjustments.

b Model 2 was adjusted for age, sex, living area, ethnicity.

c Model 3 was further adjusted for educational level, household income, marital status, and eGFR.

d *p* value for LES/HBS/HFS groups indicated *p*-trends which were calculated by including the corresponding group as a continuous variable.

### 3.3 Subgroup analysis of LES, HBS, HFS and the risk of hyperuricemia

Table 3 shows the associations of different groups of LES, HBS, and HFS, with the risk of hyperuricemia. The effect of high LES on the risk of hyperuricemia was stronger among participants aged 45~60 years (OR = 0.13, 95% CI = 0.03~0.56, *p* for interaction = 0.042), whereas that of high HFS was greater among participants living in urban areas (OR = 0.20, 95% CI = 0.10~0.38, *p* for interaction = 0.039), those with a college education or above (OR = 0.11, 95% CI = 0.04~0.28, *p* for interaction = 0.025) and those with an annual income of 20,000~50,000 CNY/year (OR = 0.12, 95% CI = 0.04~0.31, *p* for interaction = 0.007).

**Table 3:** Subgroup analyses of the associations between different levels of LES, HBS, HFS and the risk of hyperuricemia^a^.

Subgroup	LES			*p* for interaction	HBS			*p* for interaction	HFS			*p* for interaction
	Moderate	High	*p*-trend^b^		Moderate	High	*p*-trend		Moderate	High	*p*-trend	
Sex				0.242				0.325				0.895
Males	0.57 (0.24, 1.36)	0.23 (0.08, 0.64)	<0.001		1.44 (0.68, 3.08)	1.31 (0.58, 2.94)	0.763		0.59 (0.31, 1.11)	0.22 (0.10, 0.47)	<0.001	
Females	1.58 (0.16, 15.76)	0.89 (0.09, 9.03)	<0.001		–	–	–		0.71 (0.35, 1.48)	0.42 (0.19, 0.96)	<0.001	
Age group				0.042				0.156				0.072
18~	1.45 (0.29, 7.29)	0.48 (0.09, 2.65)	0.002		2.31 (0.64, 8.40)	1.58 (0.42, 5.99)	0.565		0.81 (0.31, 2.10)	0.29 (0.11, 0.80)	<0.001	
45~	0.19 (0.05, 0.69)	0.13 (0.03, 0.56)	0.047		0.81 (0.26, 2.55)	1.17 (0.37, 3.73)	0.435		0.27 (0.12, 0.61)	0.12 (0.04, 0.32)	<0.001	
60~	1.55 (0.30, 7.91)	0.76 (0.13, 4.33)	0.123		2.10 (0.46, 9.51)	1.32 (0.28, 6.26)	0.349		1.33 (0.56, 3.14)	0.92 (0.34, 2.49)	0.665	
Living area				0.392				0.158				0.039
Urban	1.28 (0.35, 4.65)	0.61 (0.16, 2.31)	0.002		1.91 (0.70, 5.22)	1.89 (0.68, 5.25)	0.503		0.38 (0.21, 0.72)	0.20 (0.10, 0.38)	<0.001	
Rural	0.36 (0.12, 1.03)	0.13 (0.03, 0.48)	0.002		1.16 (0.41, 3.30)	0.52 (0.16, 1.64)	0.035		1.22 (0.56, 2.65)	0.38 (0.14, 1.09)	0.046	
Educational level				0.188				0.652				0.025
Junior school or below	0.35 (0.11, 1.11)	0.28 (0.08, 1.01)	0.152		1.28 (0.41, 3.98)	0.99 (0.30, 3.25)	0.531		0.81 (0.39, 1.66)	0.50 (0.21, 1.20)	0.100	
High school	1.18 (0.24, 5.70)	0.34 (0.06, 2.01)	0.021		2.31 (0.49, 10.99)	1.06 (0.21, 5.40)	0.235		0.72 (0.24, 2.16)	0.62 (0.20, 1.95)	0.437	
College or above	1.06 (0.20, 5.58)	0.43 (0.08, 2.35)	0.003		1.53 (0.48, 4.86)	1.66 (0.51, 5.43)	0.480		0.38 (0.16, 0.89)	0.11 (0.04, 0.28)	<0.001	
Household income per capita (CNY/year)				0.08				0.966				0.007
<20,000	0.26 (0.08, 0.84)	0.20 (0.05, 0.77)	0.080		1.57 (0.42, 5.90)	1.09 (0.27, 4.39)	0.558		1.18 (0.47, 2.95)	1.03 (0.37, 2.88)	0.962	
20,000~	1.95 (0.37, 10.17)	0.53 (0.09, 2.99)	0.001		1.78 (0.49, 6.44)	1.24 (0.33, 4.69)	0.473		0.51 (0.23, 1.11)	0.12 (0.04, 0.31)	<0.001	
50,000~	0.77 (0.15, 3.84)	0.37 (0.07, 1.99)	0.014		1.22 (0.37, 3.98)	1.20 (0.36, 4.00)	0.897		0.37 (0.15, 0.88)	0.18 (0.07, 0.45)	<0.001	

LES, Life’s Essential 8 score; HBS, healthy behaviors score; HFS, healthy factors score; CNY, Chinese Yuan.

a Calculated based on model 3 without adjusting the corresponding subgroup factor.

b *p*-trend for LES/HBS/HFS groups was calculated by including the corresponding group as a continuous variable.

When LES, HBS and HFS were included as continuous variables (Table 4), the associations of every 10-unit increase in both LES and HFS with the risk of hyperuricemia were greater in participants who had an educational level of college or above and an annual household income of 20,000~50,000 CNY/year.

**Table 4:** Subgroup analysis of the associations between per 10-unit increase in LES, HBS, HFS and the risk of hyperuricemia^a^.

Subgroup	LES			HBS			HFS		
	OR (95% CI)	*p* value	*p* for interaction	OR (95% CI)	*p* value	*p* for interaction	OR (95% CI)	*p* value	*p* for interaction
Sex			0.745			0.659			0.376
Males	0.76 (0.63, 0.91)	<0.001		0.98 (0.86, 1.12)	0.816		0.70 (0.61, 0.80)	<0.001	
Females	0.78 (0.63, 0.96)	<0.001		0.95 (0.80, 1.12)	0.527		0.85 (0.74, 0.97)	<0.001	
Age group			0.319			0.437			0.054
18~	0.73 (0.58, 0.91)	0.006		0.96 (0.81, 1.13)	0.597		0.74 (0.63, 0.86)	<0.001	
45~	0.73 (0.56, 0.96)	0.023		1.08 (0.89, 1.32)	0.429		0.65 (0.53, 0.79)	<0.001	
60~	0.83 (0.65, 1.07)	0.147		0.89 (0.74, 1.08)	0.233		0.92 (0.78, 1.09)	0.336	
Living area			0.852			0.473			0.239
Urban	0.76 (0.64, 0.90)	0.002		1.01 (0.89, 1.15)	0.855		0.73 (0.65, 0.82)	<0.001	
Rural	0.73 (0.57, 0.93)	0.010		0.86 (0.71, 1.03)	0.098		0.82 (0.70, 0.97)	0.021	
Educational level			0.049			0.511			0.007
Junior school or below	0.90 (0.71, 1.13)	0.361		1.01 (0.85, 1.20)	0.876		0.87 (0.74, 1.02)	0.078	
High school	0.79 (0.60, 1.04)	0.090		0.88 (0.70, 1.11)	0.277		0.85 (0.70, 1.04)	0.115	
College or above	0.65 (0.52, 0.81)	<0.001		0.98 (0.83, 1.15)	0.777		0.65 (0.55, 0.76)	<0.001	
Household income per capita (CNY/year)			0.027			0.774			0.007
<20,000	0.93 (0.71, 1.23)	0.629		0.94 (0.77, 1.15)	0.571		0.94 (0.78, 1.12)	0.456	
20,000~	0.64 (0.50, 0.80)	<0.001		0.93 (0.78, 1.11)	0.473		0.67 (0.57, 0.79)	<0.001	
50,000~	0.75 (0.59, 0.94)	0.013		1.00 (0.84, 1.18)	0.978		0.72 (0.61, 0.84)	<0.001	

LES, Life’s Essential 8 score; HBS, healthy behaviors score; HFS, healthy factors score; CNY, Chinese Yuan.

a Calculated based on model 3 without adjusting the corresponding subgroup factor.

## 4 Discussion

In this study, inverse dose–response associations of the LES and its subscales of HFS with the risk of hyperuricemia in Chinese adults were observed, with 24% and 23% reductions in the risk of hyperuricemia for every 10-unit increase, respectively. Compared with those with low LES and HFS, participants with high LES and HFS were both at lower risk of hyperuricemia. Subgroup analyses revealed that these relationships were stronger among urban, middle-class, and highly educated individuals. In contrast, no significant association was observed with the subscales of the HBS.

Since the release of Life’s Essential 8 in 2022, studies have explored the association between LES and hyperuricemia among U.S. adults; however, research in Chinese populations has been limited to elderly and multiethnic groups. Evidence from U.S. studies demonstrated that participants in the high LES group had a 50%~80% reduction in the risk of hyperuricemia compared with those in the low LES group, and every 10-point increase in LES was associated with a 3%~30% lower risk of hyperuricemia (15–18). Among the few studies involving Chinese populations, Jiao et al. (19) reported a significant inverse association between LES and the risk of hyperuricemia in elderly individuals aged 60 years or above, which was supported by multiethnic Chinese data in which ethnic minorities made up half of the participants (20). Additionally, data from the Kailuan Cohort Study in China revealed that an increase in the LES reduced the risk of cardiovascular disease in hyperuricaemic individuals (25). These findings collectively indicated a link between LES and hyperuricemia, yet direct evidence from the general population, predominantly the Han Chinese population, is still lacking. As the associations observed solely in high-risk groups (such as the elderly) or specific groups may overestimate or underestimate the true impact of LES and are often not generalizable, it is thus essential to conduct research in the general population to accurately estimate population attributable risk, ensure the broad applicability of the findings and enable the government to rationally allocate public health resources.

Our findings revealed a significant association between HFS and the risk of hyperuricemia, which was consistent with the findings of previous studies, as each component of HFS has been found to be a risk factor for hyperuricemia (26–29). Nevertheless, no association was observed between HBS and the presence of hyperuricemia. In line with our findings, many studies have also shown a stronger effect for HFS, indicating a lack of association or a weak association between HBS and the incidence of hyperuricemia (18, 19). These findings may be due to the inconsistent results of two components of HBS, sleep and smoking. Two Chinese studies have shown that shorter sleep duration was associated with a greater risk of hyperuricemia (30, 31), and U-shaped or negative associations have also been reported (32, 33). For smoking, most studies have focused on gout, the final clinical consequence of hyperuricemia, and have reported complex results (34). For example, data from the Framingham Heart Study revealed that smoking was a protective factor for the incidence of gout (35), which was supported by another Singapore study in men, but no association was observed among women (36). Thus, further research to elucidate the effect of HBS is clearly warranted.

The marker of hyperuricemia, uric acid, is a metabolic product of purine catabolism, and its levels are frequently found to be elevated in individuals with cardiovascular disease (37). This may be because serum uric acid is involved in the pathophysiology of cardiovascular disease through increased oxidative stress, inflammation and apoptosis (38, 39). Another possible reason is that elevated uric acid levels and cardiovascular disease share common risk factors, such as obesity and insulin resistance. More specifically, adipose tissue in obese individuals could increase uric acid production by enhancing the activity of xanthine oxidoreductase and the pentose phosphate pathway (40). Decreased insulin sensitivity might stimulate renal tubules and thus reduce uric acid excretion and increase uric acid reabsorption (41). These findings provided mechanistic support for the relationship between uric acid and cardiovascular disease. Given that LES was established to enhance CVH, finding the inverse association between LES and hyperuricemia is unsurprising.

Strengths of this study include the investigation of the newly updated LES as well as its subscales, the examination of both linear and non-linear relationships, and the contribution to the available evidence on the effectiveness of these metrics in the general Chinese population. Hence, our findings provide insight into the role of LES in evaluating and promoting not only CVH but also hyperuricemia prevention, and may support the wider application of LES in population health policy and practice.

There are also several limitations in our study. First, our study population was drawn solely from Beijing and therefore lacked nationwide representation. Second, as a cross-sectional analysis, this study cannot definitively establish causality, even after adjustment for confounders; therefore, large-scale longitudinal studies are needed to confirm the causal relationships. Third, the use of a food frequency questionnaire in this study was limited by potential recall bias and an inability to accurately assess day-to-day variations in dietary intake. Finally, although we adjusted for multiple confounding factors, we cannot rule out residual confounding due to unmeasured or unknown variables.

In conclusion, our study demonstrated a negative association between the LES and the risk of hyperuricemia among general Chinese population in Beijing, as well as its subscale of HFS. These findings highlight the potential of LES as a public health strategy for alleviating the disease burden of hyperuricemia. Future longitudinal cohort studies are warranted to establish a causal relationship between LES and the risk of hyperuricemia and to elucidate interactions among LES components.

## Data Availability

The raw data supporting the conclusions of this article will be made available by the authors, without undue reservation.
